# Randomized Trial of 21% versus 100% Oxygen during Chest Compressions Followed by Gradual versus Abrupt Oxygen Titration after Return of Spontaneous Circulation in Neonatal Lambs

**DOI:** 10.3390/children10030575

**Published:** 2023-03-17

**Authors:** Deepika Sankaran, Evan M. Giusto, Amy L. Lesneski, Morgan E. Hardie, Houssam M. Joudi, Emily C. A. Lane, Victoria L. Hammitt, Kirstie C. Tully, Payam Vali, Satyan Lakshminrusimha

**Affiliations:** 1 Department of Pediatrics, University of California, Davis, Sacramento, CA 95817, USA; 2 Department of Stem Cell Research, University of California, Davis, Sacramento, CA 95817, USA

**Keywords:** chest compressions, inspired oxygen, neonatal resuscitation, cardiac arrest, asphyxia, hyperoxia, oxidative stress, post-resuscitation care

## Abstract

The combination of perinatal acidemia with postnatal hyperoxia is associated with a higher incidence of hypoxic-ischemic encephalopathy (HIE) in newborn infants. In neonatal cardiac arrest, current International Liaison Committee on Resuscitation (ILCOR) and Neonatal Resuscitation Program (NRP) guidelines recommend increasing inspired O_2_ to 100% during chest compressions (CC). Following the return of spontaneous circulation (ROSC), gradual weaning from 100% O_2_ based on pulse oximetry (SpO_2_) can be associated with hyperoxia and risk for cerebral tissue injury owing to oxidative stress. We hypothesize that compared to gradual weaning from 100% O_2_ with titration based on preductal SpO_2_, abrupt or rapid weaning of inspired O_2_ to 21% after ROSC or use of 21% O_2_ during CC followed by upward titration of inspired O_2_ to achieve target SpO_2_ after ROSC will limit hyperoxia after ROSC. Nineteen lambs were randomized before delivery and asphyxial arrest was induced by umbilical cord occlusion. There was no difference in oxygenation during chest compressions between the three groups. Gradual weaning of inspired O_2_ from 100% O_2_ after ROSC resulted in supraphysiological PaO_2_ and higher cerebral oxygen delivery compared to 21% O_2_ during CC or 100% O_2_ during CC followed by abrupt weaning to 21% O_2_ after ROSC. The use of 21% O_2_ during CC was associated with very low PaO_2_ after ROSC and higher brain tissue lactic acid compared to other groups. Our findings support the current recommendations to use 100% O_2_ during CC and additionally suggest the benefit of abrupt decrease in inspired oxygen to 21% O_2_ after ROSC. Clinical studies are warranted to investigate optimal oxygen titration after chest compressions and ROSC during neonatal resuscitation.

## 1. Introduction

Many newly born infants impacted by perinatal asphyxia need resuscitation immediately after birth. Previous studies have shown that even brief exposure to 100% inspired oxygen augments systemic oxidative stress and lung oxidative injury in ventilated term newborn lambs [1]. The American Academy of Pediatrics—Neonatal Resuscitation Program (AAP-NRP) has provided guidelines for preductal pulse oximetry oxygen saturation (SpO_2_) targets during resuscitation [2]. The 2020 International Liaison Committee on Resuscitation (ILCOR) guidelines advocate the use of 100% oxygen when chest compressions are indicated [3]. Owing to poor circulation during cardiac arrest in spite of chest compressions, it is challenging to accurately monitor oxygenation status using a pulse oximeter. Once return of spontaneous circulation (ROSC) is achieved, SpO_2_ can be measured and can be utilized to guide the administration of supplemental oxygen. However, there are no clear guidelines on how quickly and by how much the supplemental O_2_ needs to be titrated. Hypercapnia following asphyxia is accompanied by a rise in cerebral blood flow and reperfusion injury. On the contrary, hypocarbia following ROSC can reduce cerebral blood flow [4,5]. Use of 100% oxygen during neonatal resuscitation with chest compressions and gradual weaning of inspired oxygen based on SpO_2_ can be associated with ischemia-reperfusion compounded by hyperoxia [6] and a risk for free-radical-mediated cerebral oxidative stress and tissue injury [7].

We have previously shown the benefit of abrupt weaning of inspired oxygen to 21% oxygen from 100% oxygen following ROSC after the initial use of 100% oxygen during chest compressions in limiting cerebral hyperoxia [8]. However, we did not randomize lambs to receive 21% oxygen during chest compressions in this study. Moreover, we did not evaluate markers of oxidative stress or lactic acid in the brain tissue in the previous study.

**Figure 1:**
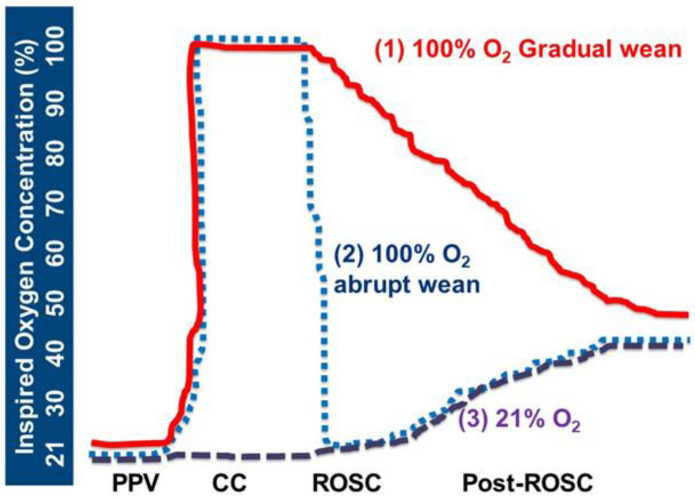
Study protocols: red line: 100% CC, gradual wean; blue dotted line: 100% CC, abrupt wean; and purple dashed line: 21% CC O_2_. PPV, positive pressure ventilation. CC, chest compressions. ROSC, return of spontaneous circulation. Post-ROSC, after return of spontaneous circulation.

We hypothesized that gradual weaning of inspired O_2_ from 100% as currently recommended by NRP (solid red line in Figure 1) following ROSC will increase cerebral oxygen delivery to the newborn brain during chest compressions and exacerbate reperfusion injury after ROSC compared to use of 21% oxygen during chest compressions (dashed purple line) and 100% oxygen during chest compressions followed by abrupt weaning to 21% (dotted blue line). Use of 21% oxygen during chest compressions will decrease cerebral oxygen delivery (hyphenated purple line). We also hypothesized that gradual weaning of inspired oxygen from 100% following ROSC as currently recommended by the NRP will increase oxidative stress to the newborn brain and exacerbate ischemia-reperfusion injury compared to abrupt weaning to 21% or use of 21% oxygen during chest compressions.

## 2. Methods

The experimental protocol was approved by the Institutional Animal Care and Use Committee (IACUC, protocol #22544) at the University of California Davis, Davis, CA, USA. This protocol, which involves a perinatal model of asphyxial arrest in term newborn lambs, has been described previously [9]. Experiments for this protocol were performed per animal ethical guidelines (ARRIVE) [10]. Time-dated near-term (139–142 days) gestation pregnant ewes were procured from Van Laningham Farm (Arbuckle, CA, USA). After an overnight fast, the ewes underwent a cesarean section under general anesthesia with intravenous (IV) diazepam, ketamine, and inhaled 2% isoflurane [9].

### 2.1. Instrumentation in Utero

The fetal lamb was partially exteriorized and the airway was intubated (4.5 mm cuffed endotracheal tube/ETT). Lung fluid was passively drained by gravity, and the ETT was occluded to prevent the entry of air during gasping. The lamb instrumentation was performed under maternal anesthesia. Additional local anesthesia was provided using subcutaneous bupivacaine infiltration. A catheter was inserted into the right carotid artery for preductal arterial blood draws and invasive blood pressure and heart rate monitoring. Another catheter was inserted into the right jugular vein for intravenous access for fluid and medication administration. A left carotid artery flow probe (Transonics, Ithaca, NY, USA) was secured to measure and record blood flow to the brain. The hemodynamics were recorded at fetal baseline. Arterial blood gases and plasma samples were also obtained.

### 2.2. Asphyxial Cardiac Arrest and Resuscitation

Fetal lambs were asphyxiated by umbilical cord occlusion [9]. After a flat line was achieved on carotid arterial pressure tracing with a heart rate of zero, the umbilical cord was clamped, the lamb was delivered and placed under a radiant warmer. A low umbilical venous catheter was placed to a depth of 2–4 cm below the skin during the period of asystole. Resuscitation was commenced after 5 min of asystole. The ETT occluder was removed and PPV was initiated using 21% oxygen with a T-piece resuscitator with peak inflation pressures of 30–35 cm H_2_O, PEEP of 5 cm H_2_O, and rate of 40 breaths per minute. An end-tidal CO_2_ detector and exhaled tidal volume monitor were connected to the ETT. The research team was not blinded to the intervention due to the nature of the study. 

Thirty seconds after the onset of PPV, chest compressions were initiated and further resuscitation was determined based on the randomization. Lambs were randomized with the help of opaque envelopes prior to starting the fetal instrumentation to the following 3 groups:

- Group 1: 100% O_2_ CC—Gradual wean: Inspired oxygen was increased to 100% during CC and resuscitation was continued per AAP-NRP guidelines. Following ROSC, attempts were made to adjust inspired oxygen down from 100% O_2_ by 5–10% every 30 sec to achieve preductal SpO_2_ per NRP guidelines. After the first 10 min, we targeted 85–95% SpO_2_ in lambs that achieved ROSC during the first 30 min after ROSC, and 90–95% beyond 30 min after ROSC until 60 min for all the study lambs.
- Group 2: 100% O_2_ CC—Abrupt wean: Inspired oxygen was increased to 100% during CC, and resuscitation was continued per NRP. After ROSC, the inspired oxygen was rapidly decreased to 21% and attempts were made to adjust inspired oxygen up by 5–10% every 30 sec to maintain preductal SpO_2_ as described above.
- Group 3: 21% O_2_ CC: Inspired oxygen of 21% was administered during chest compressions. Following ROSC, inspired oxygen was titrated up by 5–10% every 30 sec to achieve preductal SpO_2_ as described above.

After initial resuscitation, the ETT was connected to a conventional mechanical ventilator. Target PaCO_2_ was 40–60 mm Hg to allow permissive hypercapnia and to avoid hypocapnia in the setting of hypoxic ischemic encephalopathy. Hemodynamics (carotid artery blood flow, heart rate, and carotid artery blood pressure) were continuously monitored, and arterial blood samples were obtained at fetal baseline, during resuscitation, and at serial intervals after ROSC. Lambs were monitored for up to 60 min after successful ROSC and were subsequently euthanized using IV pentobarbital (Fatal-Plus, Vortech Pharmaceuticals, Dearborn, MI, USA).

Plasma samples were frozen and analyzed at the Western Metabolomics center, UC Davis for markers of oxidative stress including hypoxanthine/xanthine ratios, methionine sulfoxide/methionine ratio, and lactic acid. Similarly, oxidative stress markers were assessed from brain tissue that was collected after euthanasia. After euthanasia, frozen brain tissue was sent to Western Metabolomics center for analysis to detect the same markers of oxidative stress and lactic acid concentrations. 

### 2.3. Primary and Secondary Outcomes

Primary outcome measures were partial pressure of oxygen and cerebral oxygen delivery during chest compressions and after ROSC. 

Secondary outcome measures were changes in left carotid artery blood flow, PaCO_2_, SaO_2_, and oxidative stress markers in plasma and brain tissue as described above. 

Cerebral oxygen delivery (mL/kg/min) was calculated by multiplying carotid artery oxygen content (CaO_2_ = (1.36 × Hemoglobin in g/dL × SaO_2_%/100) + (partial pressure of O_2_ in mm Hg× 0.0031)) and left carotid artery blood flow. This is a representative measurement as it does not take into account the right carotid and vertebral arterial flow [11].

### 2.4. Sample Size Calculation

Sample size was calculated for the parameter cerebral oxygen delivery and based on prior data evaluating oxygen-weaning strategies in asphyxiated lambs [8]. We planned a study of a continuous response variable from independent control and experimental subjects with 1 control per experimental subject. If the true difference in the experimental and control means is 4 mL/kg/min (standard deviation 3 mL/kg/min) with contrast coefficients of (1,1,1) to factor the differences between groups, we need a total of 18 lambs with 6 lambs in each group to be able to reject the null hypothesis that the population means of cerebral oxygen delivery in the experimental groups (100% O_2_ CC—Abrupt wean and 21% O_2_ CC) and control (100% O_2_ CC—Gradual wean) group are equal to probability (power) of 0.97. The Type I error is 0.05. 

### 2.5. Data Collection and Analysis

Hemodynamic data were continuously monitored and recorded using BIOPAC systems data acquisition software (Goleta, CA, USA). Blood gases were analyzed using a blood gas analyzer (Radiometer ABL90 FLEX, Denmark). Categorical data were analyzed using chi-squared test or Fisher’s exact test as appropriate, and changes in parametric continuous data over time were compared using repeated measures ANOVA and post hoc analysis using Statview 5.0.1 (SAS Institute Inc., New York, NY, USA). Statistical significance was defined as *p* < 0.05.

## 3. Results

**Figure 2:**
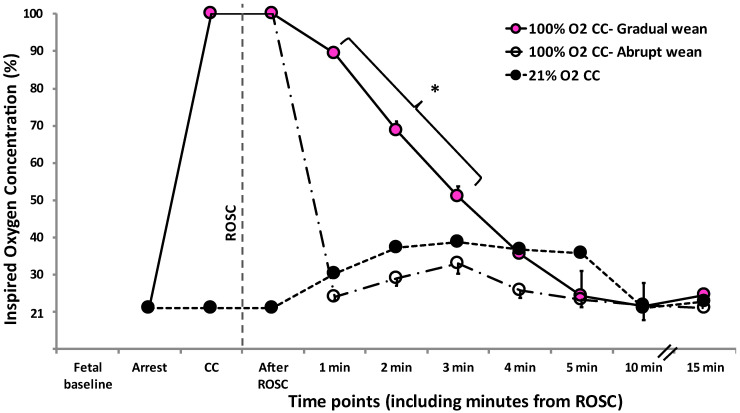
Change in inspired oxygen concentration during resuscitation and after ROSC. Per study design, the inspired oxygen concentration was significantly different between the study groups. * *p* < 0.01 ANOVA repeated measures among the 3 groups. Data presented as mean ± SEM.

Among nineteen near-term lambs that were asphyxiated, seven were randomized to 100% O_2_ CC—Gradual wean, six to 100% O_2_ CC2014Abrupt wean, and six to 21% O_2_ CC. Hemodynamic and arterial blood gas results were similar between the three groups at feta baseline (Table 1). Inspired oxygen concentration was different between the three groups based on the study design (Figure 2). All lambs achieved ROSC. However, one lamb in the 21% O_2_ CC group had a cardiac arrest after initial ROSC after the first dose of intravenous epinephrine, and chest compressions were restarted.

### 3.1. Hemodynamic and Arterial Blood Gas Parameters during Chest Compressions and after ROSC

There was no significant difference in PaO_2_, SaO_2_, and left carotid artery blood flow during chest compressions between the three study groups (Table 2). Cerebral oxygen delivery was low compared to fetal values but also not different among groups during chest compressions (Table 2). 

**Figure 3:**
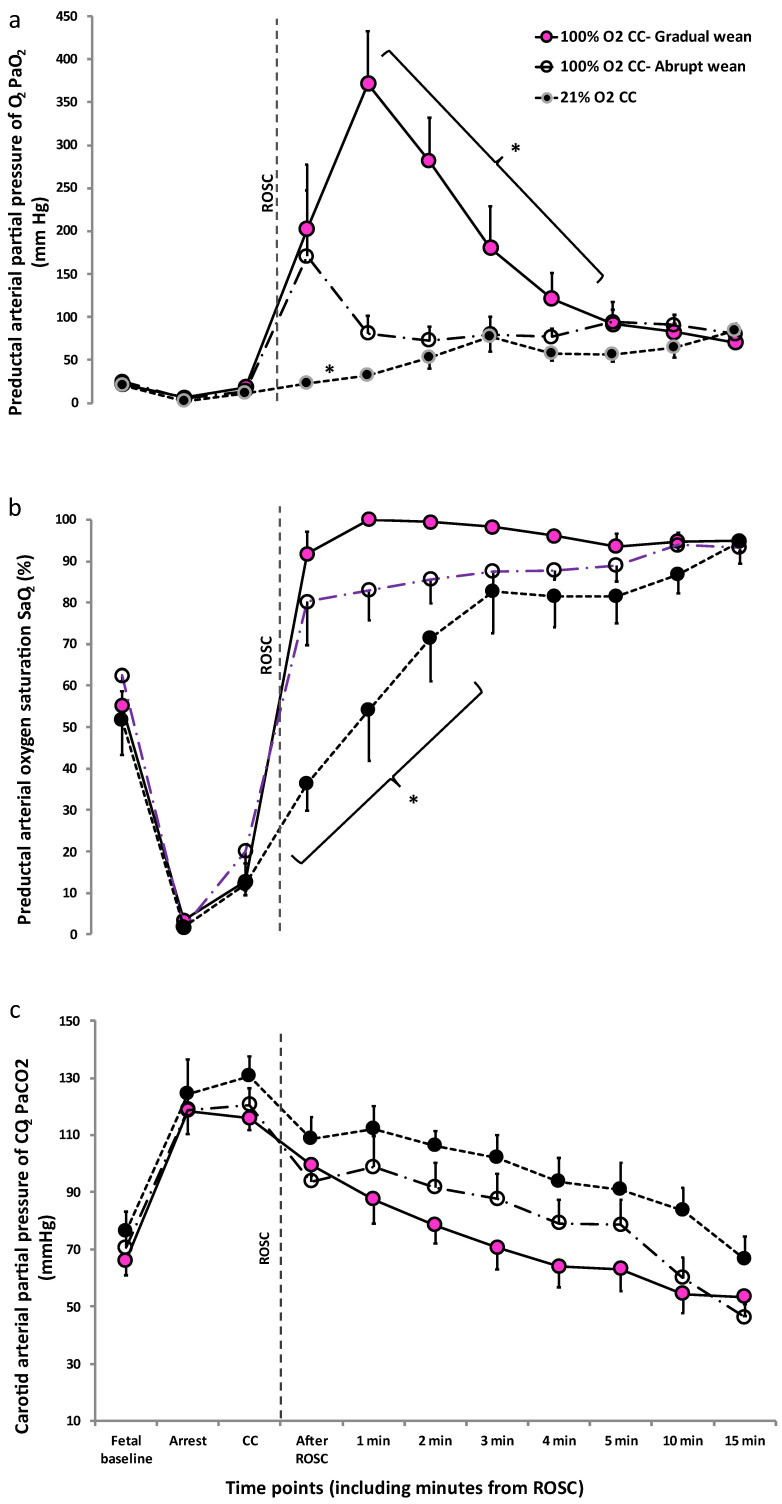
Comparison of PaO_2_ (**a**), SaO_2_ (**b**), and PaCO_2_ (**c**) between the study groups. PaO_2_ was significantly higher after ROSC, reaching supraphysiological levels with the use of 100% oxygen during chest compressions followed by gradual weaning of inspired oxygen after ROSC compared to the use of 100% O_2_ CC abrupt weaning and 21% O_2_ during chest compressions (*p* < 0.0001 100% O_2_ CC—Gradual wean vs. 21% O_2_ CC. *p* = 0.0005 100% O_2_ CC—Gradual wean vs. 100% O_2_ CC—Abrupt wean. SaO_2_ was significantly lower with the use of 21% oxygen during chest compressions compared to the other two groups (*p* = 0.03, 21% O_2_ CC vs. 100% O_2_ CC—Abrupt wean; *p*= 0.002, 21% O_2_ CC vs. 100% O_2_ CC—Gradual wean), and higher in 100% O_2_ CC—Gradual wean compared to 100% O_2_ CC—Abrupt wean (*p* = 0.03). PaCO_2_ was not significantly different between the three groups 100% O_2_ CC—Gradual wean, 100% O_2_ CC—Gradual wean, and 21% O_2_ CC by repeated measures ANOVA. * *p*< 0.05 compared to the other 2 groups. Data presented as mean ± SEM.

Following ROSC, PaO_2_ was significantly higher in 100% O_2_ CC—Gradual wean compared to the other two groups, reaching supraphysiological levels (Figure 3a).

Preductal arterial oxygen saturation (SaO_2_) was significantly higher after ROSC with 100% O_2_ CC—Gradual wean compared to 100% O_2_—Abrupt wean and 21% O_2_ CC (*p* = 0.03, Figure 3b). SaO_2_ remained very low in the 21% O_2_ CC group up to 3 min after ROSC (Figure 3b). PaCO_2_ was not different during chest compressions and after ROSC (Figure 3c).

**Figure 4:**
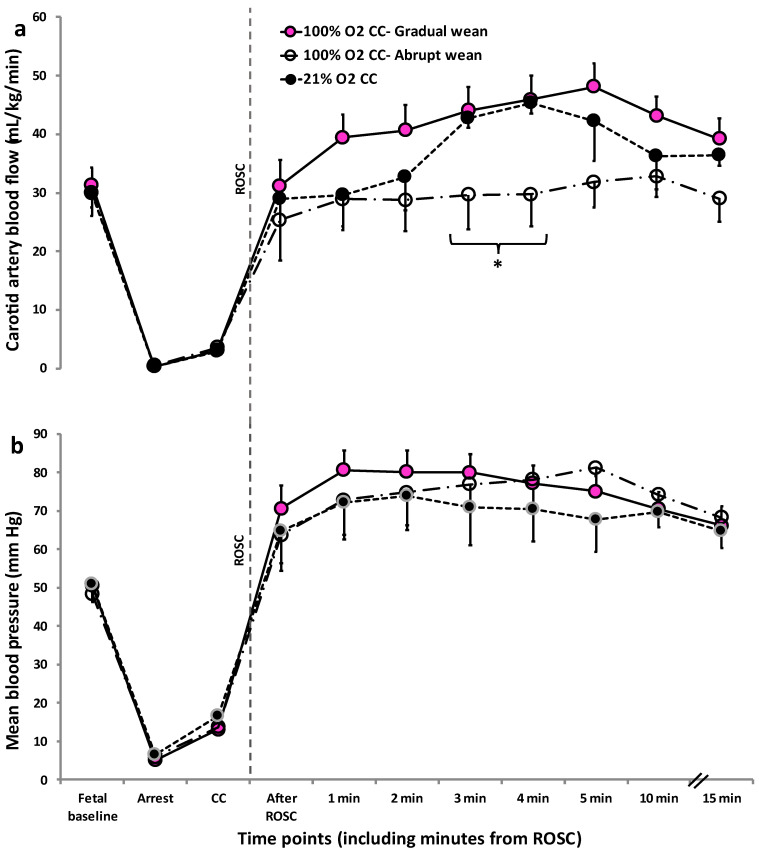
Comparison of left carotid artery blood flow (**a**) and mean blood pressure (**b**). Left carotid artery blood flow was lower and closer to fetal baseline with 100% O_2_ CC—Abrupt wean compared to 100% O_2_ CC—Gradual wean and 21% O_2_ CC but was not different between the groups by repeated measures ANOVA by 10 min after ROSC (*p* = 0.8, 0.26 and 0.38 when comparing the 3 groups). Mean blood pressures were not different between the groups by repeated measures ANOVA (*p* > 0.8). Mean blood pressures were high in all three groups when compared to fetal baseline following ROSC after chest compressions. Data presented as mean ± SEM. * *p* = 0.04 left carotid artery blood flow between 21% O_2_ CC vs. other 2 groups at 3 and 4 min after ROSC. 3.3. Comparison of cerebral oxygen delivery during chest compressions and after ROSC.

Left carotid artery blood flow was lower and closer to fetal baseline levels with 100% O_2_ CC—Abrupt wean (Figure 4a) at 3 and 4 min after ROSC but was not different between the groups by 5 and 10 min after ROSC. Mean blood pressures were not different between the groups at fetal baseline (Figure 4b). Following ROSC, mean blood pressures increased significantly compared to fetal baseline in all three groups, but were not different between 100% O_2_ CC—Gradual wean, 100% O_2_ CC—Abrupt wean, and 21% O_2_ CC groups.

**Figure 5:**
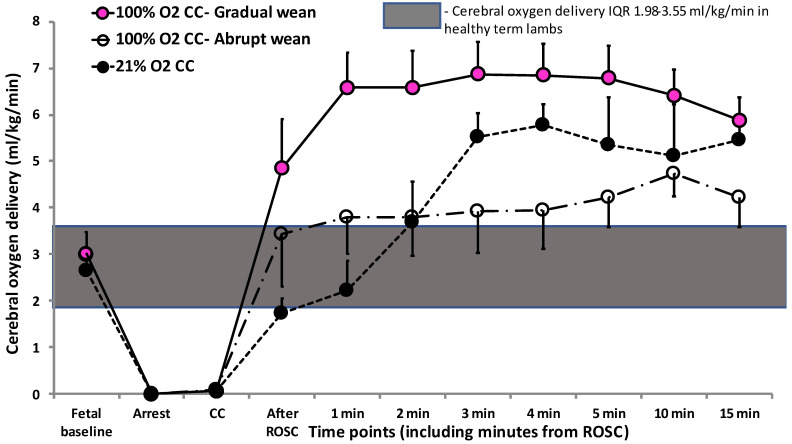
Cerebral oxygen delivery during resuscitation and after return of spontaneous circulation (ROSC). 100% O_2_—Gradual wean resulted in higher cerebral oxygen delivery reaching supraphysiological levels after ROSC compared to the 100% O_2_—Abrupt wean (*p* = 0.0001) and 21% O_2_ CC (*p* = 0.0002) groups. Data presented as mean ± SEM.

Cerebral oxygen delivery was low during chest compressions compared to fetal values. However, after ROSC, it was higher in 100% O_2_ CC—Gradual wean compared to the abrupt wean and 21% O_2_ CC groups (Figure 5), reaching supraphysiological levels within 5 min after ROSC. 

### 3.2. Comparison of Markers of Oxidative Stress in Plasma and Brain Tissue

Plasma markers of oxidative stress, including hypoxanthine/xanthine ratio, methionine sulfoxide ratio, and lactic acid, were not different between the three study groups at fetal baseline (Table 3). Although the plasma hypoxanthine/xanthine ratio was higher at 5 min after ROSC in the 100% O_2_ CC—Gradual wean group compared to the other two groups, it was not different at 10 min after ROSC. Plasma lactic acid and methionine sulfoxide/methionine ratios were not different after ROSC between the three study groups (Table 3). When oxidative stress markers were compared in brain tissue, lactic acid was higher in the 21% O_2_ CC group compared to the 100% O_2_ CC—Gradual wean and 100% O_2_ CC—Abrupt wean groups. 

## 4. Discussion

**Figure 6:**
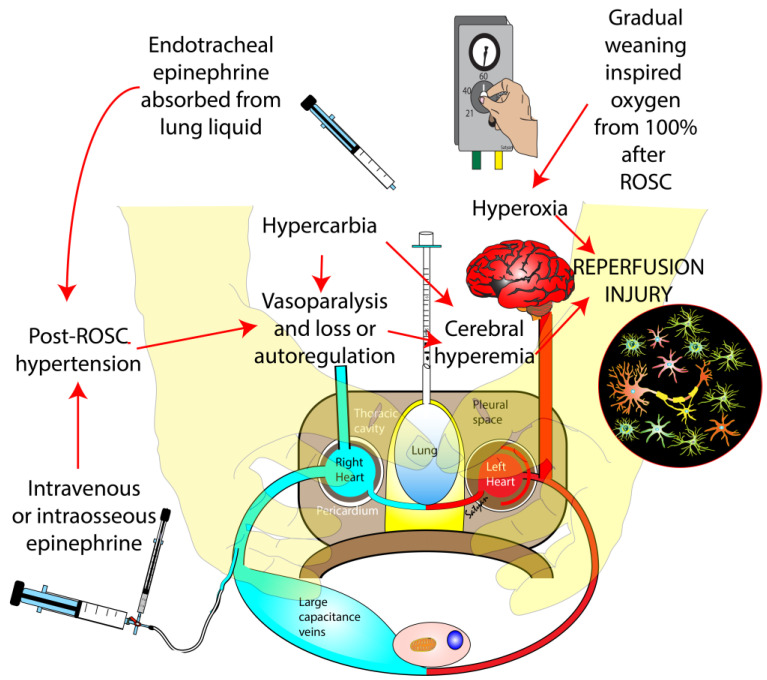
Speculative mechanisms of reperfusion injury associated with gradual weaning from 100% oxygen during ROSC following chest compressions during neonatal resuscitation. Hypercarbia associated with asphyxial injury and hypertension secondary to epinephrine use can lead to cerebral hyperperfusion. Vasoparalysis induced by asphyxia renders cerebral vasculature pressure-passive with loss of autoregulation. Hyperoxia from gradual weaning of inspired oxygen along with cerebral hyperemia may contribute to reperfusion neuronal injury. Copyright Satyan Lakshminrusimha.

The current study demonstrates the advantage of abrupt/rapid weaning of inspired oxygen to 21% immediately after ROSC following the use of 100% oxygen during chest compressions in limiting cerebral hyperoxia without resulting in systemic oxidative stress. There was no difference in the incidence of ROSC between the three study groups (100% O_2_ CC—Gradual wean, 100% O_2_ CC—Abrupt wean, and 21% O_2_ CC). Although our previous study compared weaning strategies after ROSC, we did not have a group of lambs randomized to receive 21% oxygen during chest compressions in that study [8]. Additionally, in the current study, we report lower cerebral oxygenation after ROSC with the use of 21% oxygen during chest compressions and elevated brain lactic acid compared to 100% oxygen supporting NRP recommendation to use 100% oxygen during CC. However, the current standard of gradual weaning from 100% oxygen following ROSC after chest compressions is associated with hyperoxemia in term lambs (Figure 6). 

At the cellular level, a reduction in blood flow and oxygen delivery to the brain during perinatal asphyxia initiates biochemical events that ultimately lead to anerobic metabolism and generation of lactate. This further results in energy failure and neuronal death within hours to days after asphyxia and resuscitation. The neuronal injury in the acute phase (minutes to hours) is mediated by free radicals (including oxygen free radicals) and mitochondrial damage. Generation of oxygen free radicals and reactive nitrogen species at a rate that exceeds the capacity of endogenous antioxidant systems results in oxidative stress. Due to the inherent high lipid content, high metabolic rate, and reduced activity of antioxidant enzymes, the newborn brain is vulnerable to damage incited by free radicals. After successful resuscitation and return of spontaneous circulation, newborns are exposed to a deleterious combination of hyperoxia and hyperemia (cerebral tissue reperfusion secondary to hypercarbia and possibly high blood pressure). The cerebral vasoparalysis following asphyxia leads to a loss of autoregulation and an increase in cerebral blood flow in response to hypercarbia and hypertension [5]. Epinephrine administered by the ET route is dissolved in the lung liquid and the lungs may act as a reservoir leading to an epinephrine surge and systemic hypertension following ROSC (Figure 4b and Figure 6) [12,13]. Intravenous and intraosseous epinephrine can also contribute to post-resuscitation systemic hypertension and cerebral hyperperfusion (Figure 6). Thus, a newborn surviving with hypoxic-ischemic injury is further compromised by the combination of oxidative stress and cerebral hyperemia, and reperfusion. Increased superior vena cava flow has been associated with poor outcomes in infants with HIE undergoing therapeutic hypothermia possibly due to cerebral hyperperfusion and oxidative stress [14]. This can be potentially mitigated by minimizing oxygen exposure in the post-resuscitation phase after ROSC. 

In one-day-old postnatal piglets with apnea-induced cardiac arrest [15,16], Linner et al. observed high brain tissue PaO_2_ with the use of 100% O_2_ during chest compressions and similar time to ROSC with 21% O_2_. In another study by Solevag et al., higher SpO_2_ and left ventricular oxidized glutathione ratios were reported after chest compressions with 100% O_2_ [17]. In a study comparing newly born lambs ventilated with 21% vs. 100% oxygen during chest compressions by Rawat et al., use of 100% O_2_ during chest compressions led to an increase in PaO_2_ and pulmonary blood flow immediately following ROSC compared to use of 21% O_2_ during chest compressions [6]. Cerebral oxygen delivery was significantly lower during chest compressions with both room air and 100% O_2_ comparable to fetal levels. In contrast, cerebral O_2_ delivery was significantly higher with 100% O_2_ immediately after ROSC. Our findings corroborate the results of Rawat et al. We have previously shown that gradual weaning of inspired oxygen following ROSC is associated with high PaO_2_ levels compared to abrupt weaning [8]. In this study, we randomized and compared three arms and included a third arm with 21% oxygen during chest compressions and up-titration of inspired oxygen after ROSC. In a recent meta-analysis, there was no difference in mortality or time to ROSC between 21% and 100% O_2_ during chest compressions [18]. A systemic review of 10 clinical studies comparing 21% and 100% O_2_ during resuscitation of asphyxiated term newborns (including many infants that did not need chest compressions) showed lowered mortality with 21% O_2_ [19]. There are no randomized clinical trials of 21% vs. 100% oxygen during chest compressions. Our study, for the first time, has demonstrated low cerebral oxygen delivery and increased brain tissue lactic acid (suggesting more anaerobic metabolism) with 21% oxygen use during chest compressions.

In asphyxiated term lambs, prolonged ventilation using 100% oxygen resulted in elevated brain tissue oxygen tension when compared to ventilation with 21% oxygen [20]. Koch et al. reported that brief exposure to hyperoxemia after hypoxic-ischemic insult increases secondary neuronal injury, interferes with myelination, and impairs functional recovery in rats [21]. Kapadia et al. reported that hyperoxia during the post-resuscitation phase after perinatal acidemia is associated with a higher incidence of moderate to severe hypoxic-ischemic encephalopathy [15]. Thus, it is crucial to decrease inspired O_2_ promptly to avoid hyperoxemia.

The AAP-NRP *Textbook of Neonatal Resuscitation* recommends initiating resuscitation with 21% O_2_ and increasing inspired oxygen to 100% if chest compressions are required [2]. Additionally, achieving a SpO_2_ target of 85–95% by 10 min after birth is recommended, without clear guidelines on how often and how much to titrate the inspired supplemental oxygen. We show the benefits of a rapid decrease in inspired oxygen to 21% immediately after ROSC in limiting cerebral hyperoxia when compared to the use of 21% oxygen during chest compressions and 100% oxygen during chest compressions followed by gradual weaning of inspired oxygen. Furthermore, higher brain lactic acid and a trend towards higher PaCO_2_ in the 21% O_2_ CC group are concerning for poor ventilation (likely secondary to a sustained elevation in pulmonary vascular resistance) and oxidative stress in the brain.

Analysis of oxidative stress markers in plasma and brain tissue revealed minimal differences between the three groups. A transient increase in plasma hypoxanthine/xanthine ratios was observed at 5 min after ROSC but not at 10 min after ROSC. We speculate that active weaning and titrating of inspired oxygen after ROSC in the study groups negated the initially observed differences (Table 3).

Our study has several limitations. Lambs were randomized before their delivery, thus resulting in an extra lamb in the 100% O_2_ CC—Gradual wean group due to a triplet delivery. There are species differences between lambs and humans that we have not accounted for in our study. We performed this study in a term ovine model of perinatal asphyxial cardiac arrest induced by umbilical cord compression. The response to our intervention may be different in models of asphyxial bradycardia, preterm lambs, and human newborns. However, the ovine model of asphyxial arrest is a well-established model used for investigating various interventions during neonatal resuscitation which are otherwise difficult to investigate in clinical trials due to ethical challenges in obtaining consent and recruiting newborns for randomization. We did not measure pulmonary blood flow as performing a thoracotomy can alter thoracic cage physics and mechanics during chest compressions. The perinatal lamb model closely mimics human cardiopulmonary physiology. Prior randomized trials in animal models investigating oxygen use have predominantly been performed in the postnatal piglet model after the cardiopulmonary transition has occurred [16].

## 5. Conclusions

In a perinatal term ovine model of asphyxial cardiac arrest, the use of 100% oxygen during chest compressions followed by abrupt weaning of inspired oxygen resulted in more physiological cerebral oxygen delivery and PaO_2_ compared to gradual weaning of inspired oxygen. The use of 21% oxygen during chest compressions resulted in suboptimal cerebral oxygen delivery and high lactic acid levels in brain tissue. These findings support the current NRP recommendations of using 100% oxygen during chest compressions, as the use of 21% oxygen is associated with hypoxemia and hypercarbia during chest compressions and after ROSC, respectively. Weaning abruptly to 21–30% oxygen from 100% soon after ROSC following chest compressions might potentially minimize reperfusion injury. Clinical trials investigating oxygenation, hemodynamics, and neurodevelopmental outcomes in term neonates comparing different oxygenation strategies during neonatal chest compressions and after ROSC are warranted.

## Figures and Tables

**Table 1:** Fetal baseline hemodynamic and arterial blood gas parameters, and incidence of return of spontaneous circulation.

Parameter	100% O_2_ CC—Gradual Wean (*n* = 7)	100% O_2_ CC—Abrupt Wean (*n* = 6)	21% O_2_ CC (*n* = 6)
Weight (kg)	3.5 ± 0.6	3.6 ± 0.6	3.7 ± 0.6
Gestational Age (days)	140 ± 1.5	139.7 ± 0.5	139.7 ± 1.4
Sex (F:M)	4:3	5:1	5:1
Hemoglobin, g/dL	12.0 ± 1.4	11.4 ± 1.0	13.4 ± 2
pH	7.24 ± 0.10	7.18 ± 0.08	7.19 ± 0.10
PaCO_2_ (mm Hg)	66 ± 14	71 ± 11	76 ± 16
PaO_2_ (mm Hg)	22 ± 6	25 ± 3	20 ± 8
Cerebral oxygen delivery (mL/kg/min)	3.0 ± 1.3	2.9 ± 0.7	2.7 ± 1.1
Lactate (mmol/L)	3.5 ± 2.2	2.5 ± 1.7	3.1 ± 2.6
Mean Carotid Artery Blood Flow (mL/kg/min)	31.3 ± 8.0	30.1 ± 6.4	29.7 ± 9.0
Duration of Asphyxia until Asystole (min)	12.5 ± 2.9	15.1 ± 2.3	16.5 ± 4.4
Incidence of ROSC *n* (%)	7 (100)	6 (100)	5 (83.3)
Number of epinephrine doses *n* (ROSC or not)	1 dose in 7 lambs (ROSC)	1 dose in 6 lambs (ROSC)	1 dose in 5 lambs ROSC) 4 doses in 1 lamb (ROSC followed by rearrest)

Data presented as mean ± standard deviation. Categorical variables were not different by chi-square test or Fisher’s exact test as appropriate between the three groups. Continuous variables were not different by repeated measures ANOVA between the three groups. PaCO_2_ = arterial carbon dioxide pressure; PaO_2_ = arterial oxygen pressure; SpO_2_ = oxygen saturation; ROSC, return of spontaneous circulation.

**Table 2:** Comparison of oxygenation during chest compressions.

Parameter	100% O_2_ CC—Gradual Wean (*n* = 7)	100% O_2_ CC—Abrupt Wean (*n* = 6)	21% O_2_ CC (*n* = 6)
PaO_2_ (mm Hg)	18.5 ± 5.5	13.7 ± 11.0	10.7 ± 4.3
Mean Carotid Artery Blood Flow (Q_CA_, mL/kg/min)	3.1 ± 0.9	3.6 ± 1.8	2.8 ± 0.8
Cerebral oxygen delivery (mL/kg/min)	0.07 ± 0.07	0.08 ± 0.07	0.06 ± 0.02

Data presented as mean and standard deviation. There were no significant differences between the three groups by ANOVA.

**Table 3:** Comparison of markers of oxidative stress and lactic acid from plasma and brain tissue samples.

Parameter	100% O_2_ CC—Gradual Wean (*n* = 7)	100% O_2_ CC—Abrupt Wean (*n* = 6)	21% O_2_ CC (*n* = 6)
Plasma markers			
Hypoxanthine/xanthine ratio Fetal Baseline	26 ± 16	26 ± 14	17 ± 12
5 min after ROSC	40 * ± 8	25 ± 6	24 ± 10
10 min after ROSC	31 ± 16	28 ± 15	24 ± 8
Methionine sulfoxide/methionine ratio			
Fetal baseline	5.5 ± 1.5	5.2 ± 0.1	5.4 ± 1.2
5 min after ROSC	6.7 ± 1.2	5.5 ± 1.1	5.8 ± 1.6
10 min after ROSC	6.6 ± 2.0	8.7 ± 1.0	7.6 ± 2.5
Lactic acid (×10^5^)			
Fetal baseline	8.7 ± 0.9	8.5 ± 2.2	13.9 ± 11.4
5 min after ROSC	39.4 ± 5.2	29.6 ± 9.4	32.4 ± 8.4
10 min after ROSC	37.5 ± 7.5	33.2 ± 2.6	30.6 ± 12.3
Brain markers			
Brain hypoxanthine/xanthine ratio	24.2 ± 35.2	9.2 ± 1.9	10.6 ± 2.1
Brain methionine sulfoxide/methionine ratio	2.2 ± 1.1	1.3 ± 0.1	1.2 ± 0.3
Brain lactic acid (×10^5^)	7.3 ± 1.7	6.3 ± 0.7	9.3 * ± 2

Data presented as mean and standard deviation. * *p* < 0.05 compared to the other two groups.

## Data Availability

The data presented in this study are available in this article.
